# The Cellular Prion Protein Negatively Regulates Phagocytosis and Cytokine Expression in Murine Bone Marrow-Derived Macrophages

**DOI:** 10.1371/journal.pone.0102785

**Published:** 2014-07-24

**Authors:** Min Wang, Deming Zhao, Yang Yang, Jin Liu, Jin Wang, Xiaomin Yin, Lifeng Yang, Xiangmei Zhou

**Affiliations:** State Key Laboratories for Agrobiotechnology, Key Lab of Animal Epidemiology and Zoonosis, Ministry of Agriculture, National Animal Transmissible Spongiform Encephalopathy Laboratory, College of Veterinary Medicine, China Agricultural University, Beijing, China; University of Melbourne, Australia

## Abstract

The cellular prion protein (PrP^C^) is a glycosylphosphatidylinositol (GPI)-anchored glycoprotein on the cell surface. Previous studies have demonstrated contradictory roles for PrP^C^ in connection with the phagocytic ability of macrophages. In the present work, we investigated the function of PrP^C^ in phagocytosis and cytokine expression in bone marrow-derived macrophages infected with *Escherichia coli*. *E. coli* infection induced an increase in the *PRNP* mRNA level. Knockout of PrP^C^ promoted bacterial uptake; upregulated *Rab5*, *Rab7*, and *Eea1* mRNA expression; and increased the recruitment of lysosomal-associated membrane protein-2 to phagosomes, suggesting enhanced microbicidal activity. Remarkably, knockout of PrP^C^ suppressed the proliferation of internalized bacteria and increased the expression of cytokines such as interleukin-1β. Collectively, our data reveal an important role of PrP^C^ as a negative regulator for phagocytosis, phagosome maturation, cytokine expression, and macrophage microbicidal activity.

## Introduction

Phagocytosis of pathogens initiates the innate immune response [1], [2]. Macrophages rely heavily on phagocytosis and subsequent degradation of microbes to help clear the invading pathogens [3]. The initial stage of the elimination process is the internalization of particles into a plasma membrane-derived vacuole known as the phagosome. Nascent phagosomes lack the ability to kill pathogens and degrade ingested targets; these properties are acquired during the process of phagosome maturation [4]. After internalization, targets are delivered from phagosomes to lysosomes for degradation [5]. The low-molecular-weight GTPases *Rab5* and *Rab7*, which govern the fusion of phagosomes with early and late endosomes, are associated with phagosome maturation [2], [6]. Additionally, early endosome antigen 1 (*Eea1*), a *Rab5* effector, is present in very early phagosomes but disappears rapidly during maturation of the organelle [7]. Late phagosomes acquire markers such as lysosomal-associated membrane protein (LAMP)-1 and LAMP-2, which are required for acquisition of *Rab7* and microbicidal properties [8]. These markers are important for phagosome fusion and maturation.

In addition to particle and pathogen internalization, activated macrophages initiate cytokine secretion, which is essential for host defense [2], [9]. Studies have demonstrated that failure to regulate cytokine secretion may induce a pathological state; indeed, excessive levels of tumor necrosis factor (TNF) and interleukin (IL)-6 lead to chronic inflammation [10], [11]. Therefore, regulatory control of phagocytosis is essential to limit damage to the host during pathogen clearance [12], and negative regulation of phagocytic activity may provide protection against pathological phagocytosis. Performing further studies on the specific signal transduction components that negatively regulate phagocytosis is essential.

Preliminary experiments have shown that cellular prion protein (PrP^C^) may play an important role in phagocytosis [13], [14]. PrP^C^ is a glycosylphosphatidylinositol (GPI)-anchored glycoprotein encoded by a specific prion protein gene (*PRNP*) [15]. It is expressed mainly in the central nervous system (CNS) but is also expressed in other cell types, including macrophages [14], [16]. PrP^C^ can be conformationally converted into PrP^SC^, which accumulates in the brain in prion disease [17]. Although a great deal has been learned about PrP^SC^ and its role in prion propagation, the physiological functions of PrP^C^ remain unclear. Numerous studies have suggested that PrP^C^ may have protective functions, including providing protection against apoptotic and oxidative stress, facilitating cellular uptake or binding of copper ions, promoting transmembrane signaling, and participating in the formation and maintenance of synapses [18]. PrP^C^ is also necessary for neuronal survival and maintenance of the myelin sheath around peripheral nerves [19], [20]. Additionally, although numerous reports have revealed a relationship between PrP^C^ and the phagocytic ability of different cell lines following ingestion of various particles, the results are conflicting. Studies have supported that *Rab7a* interacts with PrP^C^ and that endosomal compartments are involved in the trafficking and regulation of PrP^C^ [21]; however, further studies are required to elucidate the specific signaling mechanisms mediating the important roles of PrP^C^ in phagocytosis.

Therefore, in this study, we sought to investigate the role of PrP^C^ during phagosome formation and maturation, and we hypothesized that PrP^C^ may exert an important protective effect against internalized particles or pathogens.

## Materials and Methods

### Antibodies

The mouse monoclonal PrP antibody AH6 and rabbit anti-mouse β-actin antibody were purchased from Santa Cruz Biotechnology (Santa Cruz, CA, USA). The rabbit polyclonal anti-LAMP2 antibody was obtained from ProteinTech Group (Chicago, USA). The secondary antibodies, horseradish peroxidase-conjugated Affinipure goat anti-rabbit IgG, horseradish peroxidase-conjugated Affinipure goat anti-mouse IgG and rhodamine-conjugated Affinipure goat anti-rabbit IgG were purchased from Zhongshan Golden Bridge Biological Technology (Beijing, China).

### Enhanced green fluorescent protein (EGFP)-*Escherichia coli* preparation

The EGFP sequence from the pEGFP-N1 vector was cloned and inserted into the PET28a vector, yielding the recombinant plasmid PET28a-EGFP. This plasmid was transformed into DH5a competent cells for amplification (TransGen Biotech, Beijing, China). After incubation at 37°C for 10 h, the recombinant plasmid was isolated and transformed into BL21 (DE3) plyss competent cells (TransGen Biotech). When the OD_600_ of the culture reached 0.4–0.6, 0.5 mM isopropylthio-β-d-galactopyranoside (IPTG, ICN Pharmaceuticals, CA, USA) was added to induce expression of the EGFP protein. EGFP-*E. coli* was then plated on LB plates, and after 10 h, the number of colony-forming units (CFU) was determined and used for subsequent calculation of the EGFP-*E. coli* count per milliliter for other experiments.

### Primary cell cultures

Bone marrow-derived macrophages (BMDMs) were derived from bone marrow cells extracted from the femurs and tibiae of 6- to 8-week-old female ZrchI type PRNP^−/−^ mice [22] and wild-type C57BL/6 mice (Vital River Laboratory Animal Technology, Beijing, China). The mice were bred under strict specific pathogen-free conditions. All of the animal experiments were conducted in accordance with the guidelines of Beijing Municipality on the Review of Welfare and Ethics of Laboratory Animals approved by the Beijing Municipality Administration Office of Laboratory Animals (BAOLA). After euthanasia, the mice were immersed in 75% ethanol for 3 min. Then the femurs and tibiae were dissected using sterile scissors, and muscles connected to the bones were also removed. After three washes in sterile RPMI 1640 (Gibco, Grand Island, NY, USA), both epiphyses were removed using sterile scissors and forceps. The bones were flushed with a syringe filled with RPMI 1640 to extrude bone marrow into a 15 mL sterile polypropylene tube. The fresh bone marrow cells were cultured as previously described [23], with the following modifications. The cells were differentiated in a humidified incubator at 37°C with 5% CO_2_ in bone marrow differentiation medium, which was composed of RPMI 1640 supplemented with 10% heat-inactivated fetal bovine serum (FBS, Gibco, Grand Island, NY, USA), 100 µg/mL streptomycin, 100 U/mL penicillin (Gibco), and 10 ng/mL macrophage colony-stimulating factor (M-CSF, Peprotech Asia, Rehovot, Israel). Seven days later, the cells were detached from the dishes, counted, reseeded, and cultivated in tissue culture plates overnight before any further experimental procedures. For regular culture, BMDMs were grown in RPMI 1640 medium containing 10% heat-inactivated FBS, 100 µg/mL streptomycin, 100 U/mL penicillin, and 2 ng/mL M-CSF.

### Phagocytosis assays

Macrophages were plated on glass coverslips at a density of 2×10^5^ cells/well in 24-well plates overnight. EGFP-*E. coli* organisms were washed in phosphate-buffered saline (PBS; Hyclone, Logan, UT, USA) twice and then resuspended at the appropriate concentration in RPMI 1640 without serum or antibiotics. Before infection, cells were washed twice in PBS to eliminate the effects of Fc and/or complement receptors. BMDMs were infected with EGFP-*E. coli* at a multiplicity of infection (MOI) of 10 and incubated at 37°C in the presence of 5% CO_2_. After 30 or 60 min, the macrophages were washed in cold PBS three times to stop the uptake of additional bacteria and remove extracellular bacteria. The cells were fixed with 4% paraformaldehyde (Solarbio Science & Technology Co., Beijing, China) and the nuclei were stained with DAPI staining solution (Beyotime Institute of Biotechnology, Shanghai, China). The phagocytic ability of the macrophages was examined by fluorescence microscopy. In each experiment, at least 300 macrophages from triplicate wells were counted. The results were expressed as the phagocytic index (PI), calculated using the following equation: PI = percentage of cells containing *E. coli*×mean number of *E. coli* organisms per cell.

### Quantitative real-time polymerase chain reaction (qPCR)

An EASYspin Plus Cell/Tissue RNA Isolation Kit (Aidlab Biotechnologies Co., Beijing, China) was used to extract total RNA from adherent macrophages. Reverse transcription was performed using a RevertAid First-Strand cDNA Synthesis Kit (Fermentas, Vilnius, Lithuania) according to the manufacturer’s instructions. Based on previous studies [15], β-actin was selected as the internal control. qPCR was performed using an ABI Vii7 fluorescence detection system (Applied Biosystems, Foster City, CA, USA) with the primer pairs listed in Table 1, using an annealing temperature of 52°C. qPCR data were analyzed using the comparative C_T_ method (2^−ΔΔCT^) [24]. All samples were analyzed in triplicate.

**Table 1:** Primers used for qPCR.

Gene	Forward primer sequences (5′-3′)	Reverse primer sequences (5′-3′)
*PRNP*	GAGAACTTCACCGAGACC	GATGAGGAGGATGACAGG
*Rab5a*	CTGGTTCTTCGCTTTGTGA	ACTATGGCTGCTTGTGCTC
*Rab7*	GGCTTCACAGGTTGGAC	GGCTTGGCTTGGAGATTG
*Eea1*	GTGGCAGTCTAGTCAACG	CTTCGCCTTTAAGACACCTC
*TNF-α*	GCGGTGCCTATGTCTCAG	CACTTGGTGGTTTGCTACG
*IL-1β*	CAGGCTCCGAGATGAACAA	CCCAAGGCCACAGGTATTT
*IL-6*	TTGCCTTCTTGGGACTGAT	TTGCCATTGCACAACTCTTT
*β-actin*	GATCATTGCTCCTCCTGAGC	AAAGGGTGTAAAACGCAGC

### Bacterial proliferation assay

BMDMs were plated in 24-well plates and infected with *E. coli* at a ratio of 1∶10 at 37°C for 1 h. The cells were then washed three times with PBS, and RPMI 1640 with 10 ng/mL gentamicin (Sigma-Aldrich, St. Louis, Missouri, USA) was added for 30 min (0-h time point) or for an additional 1 h and 2 h. The infected macrophages were then washed three times, lysed with a solution of 1% Triton X-100 (Amresco, Solon, Ohio, USA) in sterile water at 37°C for 20 min, and shaken vigorously. The lysates were diluted, plated on LB agar plates, and incubated for 10 h at 37°C. The colonies were counted and expressed as CFU. The proliferation index (i.e., the total number of divisions divided by the number of bacteria that underwent division) was also calculated. Infections were completed in triplicate.

### Western blotting

After the cells were infected with *E. coli* for 1 h, the cell culture medium was discarded. The cells were then lysed immediately in ice-cold lysis buffer (Beyotime Institute of Biotechnology, Shanghai, China) containing protease and phosphatase inhibitors, and the total cell lysates were separated by sodium dodecyl sulfate-polyacrylamide gel electrophoresis (SDS-PAGE) on 12% gels, followed by transfer to 0.45-µm polyvinylidene fluoride (PVDF) membranes (Millipore, Billerica, USA). Nonspecific binding sites were blocked with 5% fat-free dried milk in Tris-buffered saline with Tween-20 (TBST) for 1 h at 37°C. The membranes were probed overnight at 4°C with the PrP antibody, β-actin antibody, and LAMP2 antibody. After the blots were washed with TBST three times, they were incubated with the respective secondary antibodies, horseradish peroxidase-conjugated Affinipure goat anti-mouse IgG and horseradish peroxidase-conjugated Affinipure goat anti-rabbit IgG (1∶5000), followed by enhanced chemiluminescence detection on an imaging system (Versadoc; Bio-Rad, Hercules, CA, USA).

### Immunofluorescence and confocal microscopy

Macrophages were plated on glass coverslips at a density of 2×10^5^ cells/well in 24-well plates and infected with EGFP-*E. coli*. One hour later, the cells were washed with PBS and fixed for 30 min with 4% paraformaldehyde. After fixation, cells were permeabilized with 0.3% Triton X-100. Cells were then blocked with 1% bovine serum albumin (BSA, Amresco, Solon, Ohio, USA) for 1 h at room temperature and incubated overnight with the rabbit polyclonal anti-LAMP2 antibody at 4°C. After washing in PBS, the rhodamine-conjugated Affinipure goat anti-rabbit IgG was added, and the cells were incubated for 1 h at 37°C in a humidified black box. The cells were then labeled with DAPI, mounted on slides, and observed under confocal microscopy. Negative controls were incubated without the primary antibody.

### Statistical analyses

All assays were independently performed three times. The results have been expressed as mean ± standard deviation (SD). Statistical significance was assessed using unpaired two-tailed Student’s *t*-tests. SPSS software (Chicago, IL, USA) was used for the statistical analysis. Differences were considered significant when *p*<0.05.

## Results

### Infection of BMDMs with *E. coli* affected the mRNA expression of *PRNP*

**Figure 1:**
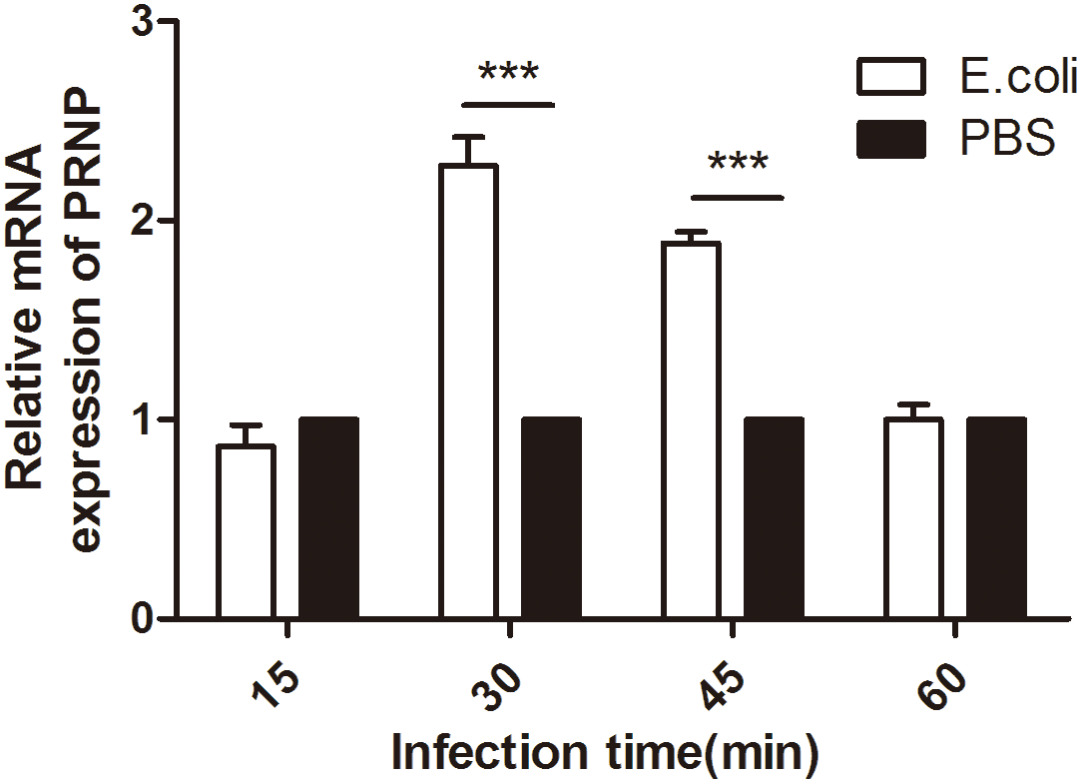
Quantitative PCR analysis of the effects of *E. coli* infection on *PRNP* mRNA levels in macrophages collected from wild-type mice. Murine bone marrow-derived macrophages were exposed to *E. coli* at an MOI of 10. Total RNA was collected at the indicated times, reverse transcribed into cDNA, and subjected to qPCR analysis. The expression of *PRNP* at each time point is expressed as the fold change relative to *PRNP* mRNA levels in control cells exposed to PBS only. Data are the mean ± SD of triplicate samples. **p≤0.01, ***p≤0.001.

BMDMs from wild-type C57BL mice were infected with EGFP-*E. coli* at an MOI of 10, and the mRNA expression of *PRNP* was examined. *PRNP* mRNA expression varied with time, and a significant increase in *PRNP* expression was observed at 30 and 45 min relative to the control (Figure 1).

### Knockout of *PRNP* promoted phagocytosis

**Figure 2:**
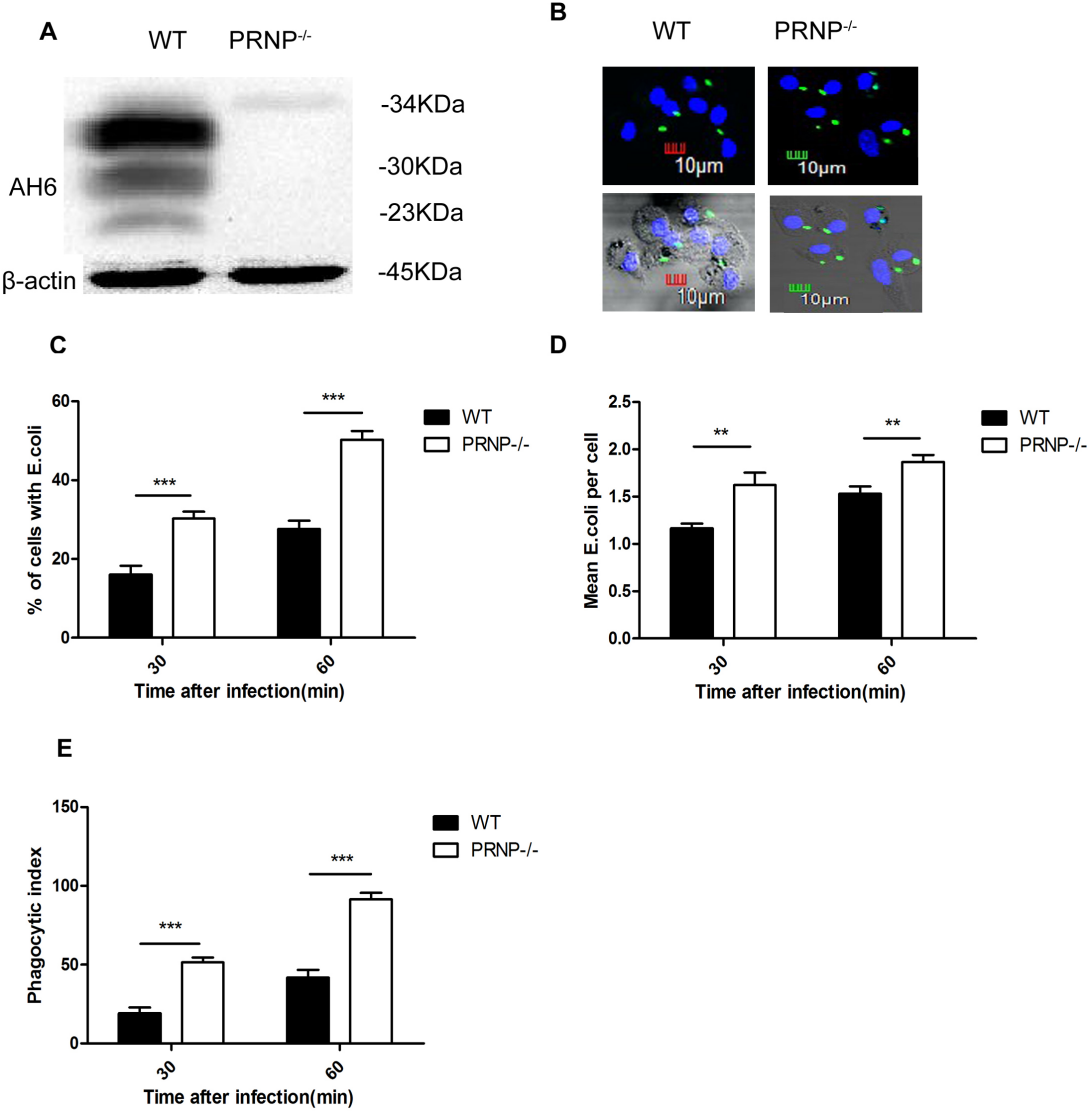
BMDMs from PRNP^−/−^ mice exhibited greater phagocytic activity than those from wild-type (WT) mice. Total proteins were collected from BMDMs and analyzed by western blotting with anti-PrP antibodies (AH6). The expected glycoforms of PrP^C^ were observed in WT samples, whereas no bands were observed in PRNP^−/−^ samples (A). Phagocytosis assays were performed using EGFP-*E. coli* (green). Cells were fixed and stained with DAPI (blue) to detect chromosomal DNA, and fluorescence microscopy was used to image the stained cells. The micrographs for 60 min are shown; the cell profile was observed through phase-contrast microscopy. (B). The percentage of cells containing *E. coli* (C), the mean count of *E. coli* per cell (D), and the phagocytic index (E) are shown. Data represent at least three independent experiments. **p≤0.01, ***p≤0.001.

Next, we examined the expression of PrP^C^ in BMDMs from PRNP^−/−^ and wild-type mice. As expected, no PrP^C^ expression was observed in PRNP^−/−^ samples (Figure 2A). To determine the role of PrP^C^ in phagocytic activity, BMDMs from PRNP^−/−^ and wild-type mice were infected with EGFP-*E. coli* for 30 and 60 min, and the PI was then assessed as a measure of the phagocytic capacity. All of the EGFP-*E. coli* was within BMDMs (Figure 2B). The percentage of cells containing *E. coli*, the mean number of *E. coli* organisms per cell, and the PI were significantly higher in BMDMs from PRNP^−/−^ mice compared to those from wild-type mice (Figure 2B, C, D, E). Furthermore, the CFU assay below gave the same result.

### Knockout of *PRNP* enhanced phagosome maturation

**Figure 3:**
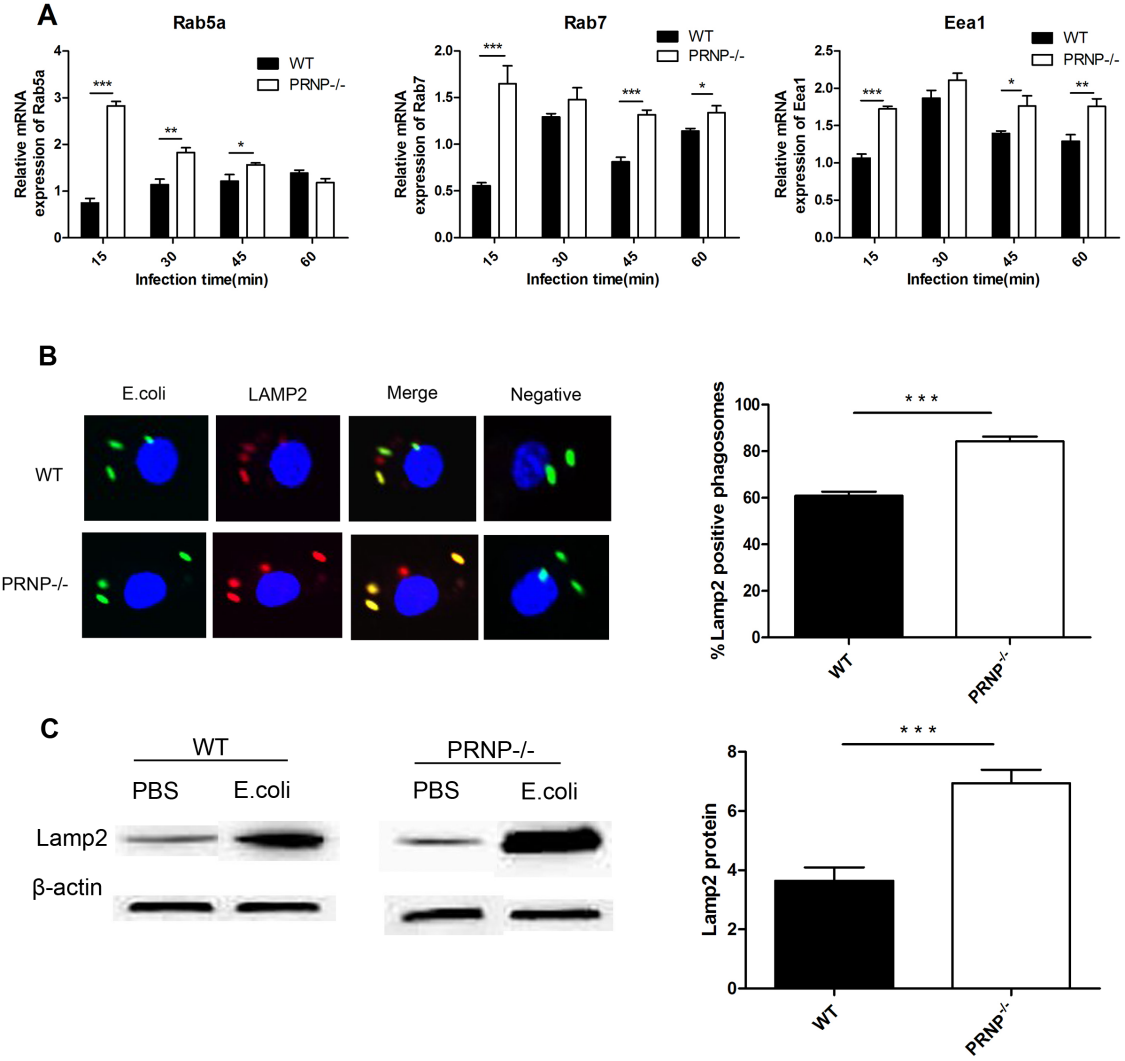
Knockout of *PRNP* regulated phagosome maturation. (A) qPCR analysis of the effects of PrP^C^ on the expression of *Rab5a*, *Rab7*, and *Eea1*. Macrophages from PRNP^−/−^ and wild-type (WT) mice were incubated with *E. coli* at 10 MOI for the indicated times. Total mRNA was isolated and reverse transcribed. The expression levels of the three markers were analyzed at each time point and are expressed as the fold change relative to the mRNA level in control cells exposed to PBS only. (B) Murine BMDMs were infected with EGFP-*E. coli* (green) at 10 MOI. Confocal fluorescence microscopy was used to image LAMP2 staining (red) in PRNP^−/−^ and WT macrophages at 1 h. Recruitment was quantified (right panel). (C) Western blot analysis of LAMP2 expression after infection for 1 h. Data represent at least three independent experiments. *p≤0.05, **p≤0.01, ***p≤0.001.

To further explore the role of *PRNP* in macrophage phagocytosis, we investigated the role of *PRNP* in phagosome maturation. Knockout of *PRNP* resulted in increased *Rab5a*, *Rab7*, and *Eea1* mRNA levels and led to an increase in the recruitment of LAMP2 to phagosomes. *Rab5a* mRNA expression was significantly higher in PRNP^−/−^ macrophages than in wild-type macrophages at 15, 30, and 45 min. Additionally, mRNA expression of *Rab7* and *Eea1* was significantly higher in PRNP^−/−^ macrophages than in wild-type macrophages at 15, 30, 45, and 60 min (Figure 3A). Confocal immunofluorescence microscopy showed that recruitment of LAMP2 to phagosomes containing EGFP-*E. coli* increased in PRNP^−/−^ macrophages following incubation with EGFP-*E. coli* for 1 h (Figure 3B). Additionally, densitometry analysis of western blotting data revealed that LAMP2 expression in PRNP^−/−^ macrophages was almost twice that in wild-type macrophages (Figure 3C).

### Knockout of *PRNP* suppressed the proliferation of internalized bacteria

**Figure 4:**
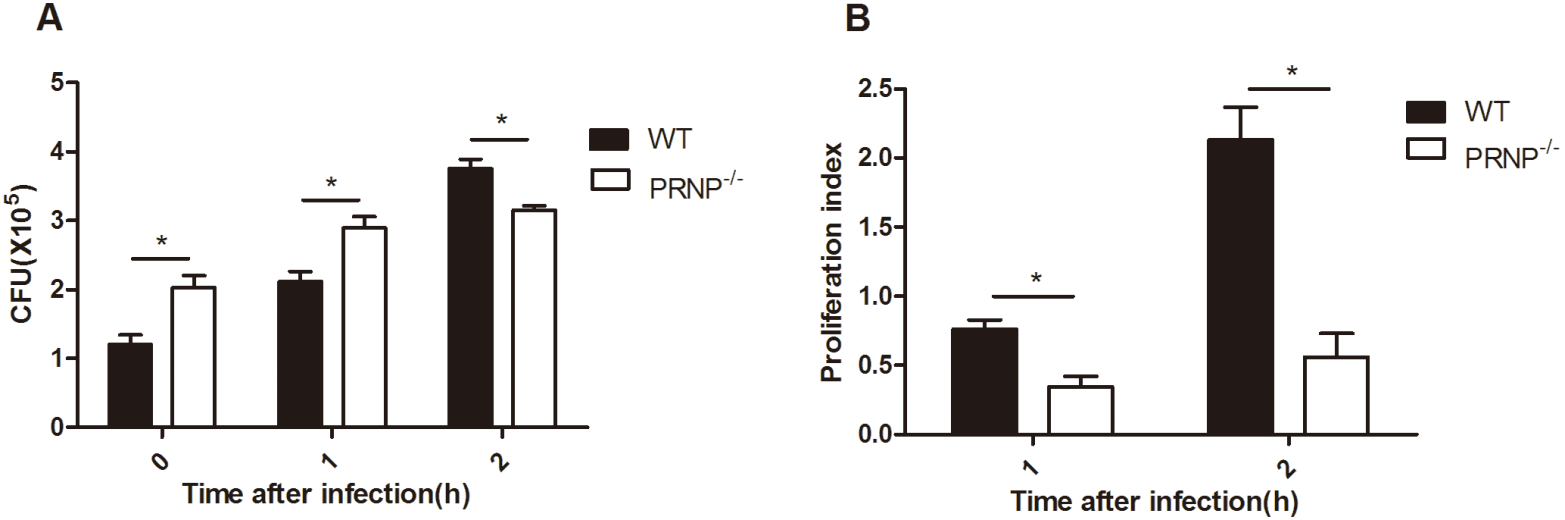
Survival of internalized *E. coli* in macrophages. (A) PRNP^−/−^ and wild-type (WT) macrophages were incubated with *E. coli* (MOI of 10) for 1 h. After washing, cells were incubated with RPMI-1640 containing gentamicin for 30 min (0-h time point) or for an additional 1–2 h. After each time point, cells were washed and lysed. Live bacteria in the lysates were counted after inoculation on LB agar plates. (B) The proliferation index at the 1-h and 2-h time points is shown. Data are representative of at least three independent experiments. *p≤0.05, **p≤0.01, ***p≤0.001.

Given that knockout of *PRNP* affected phagocytosis and phagosome maturation, we attempted to elucidate the role of *PRNP* in killing intracellular pathogens and in cytokine secretion. Macrophages were allowed to ingest *E. coli* for 1 h at 37°C and were then treated with gentamicin for various durations. The survival of *E. coli* engulfed by macrophages was analyzed by CFU enumeration. An increase in the number of CFUs was observed from 0 to 2 h. At the 0- and 1-h time points, the number of CFUs from PRNP^−/−^ macrophages was significantly higher than that from wild-type macrophages, whereas the opposite effect was observed at the 2-h time point (Figure 4A). However, the proliferation index of PRNP^−/−^ macrophages was much lower than that of wild-type macrophages (Figure 4B).

### Knockout of *PRNP* increased the expression of pro-inflammatory cytokines

**Figure 5:**
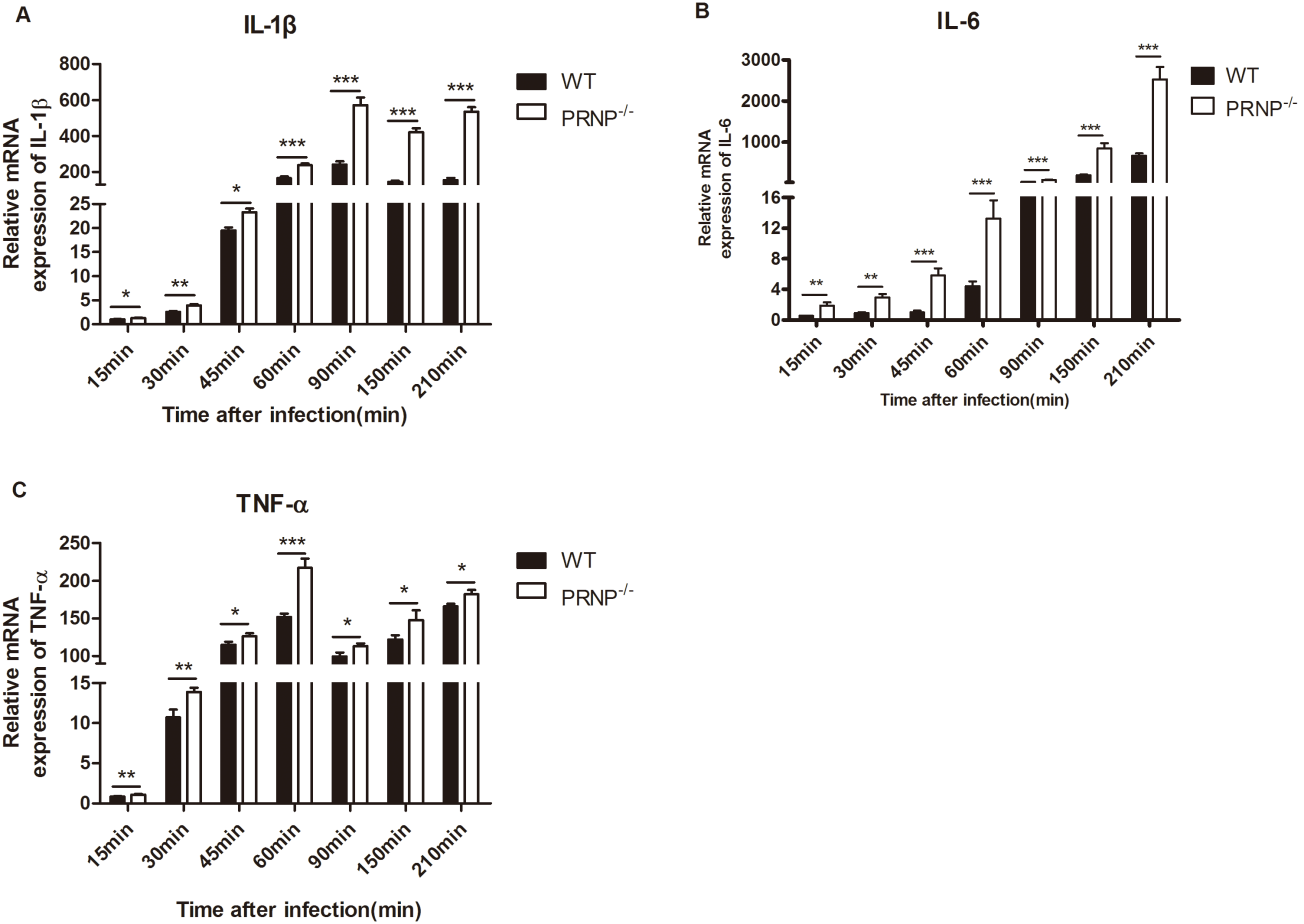
mRNA expression of pro-inflammatory cytokines. (A, B, and C) Total RNA was collected, reverse transcribed to cDNA, and analyzed by qPCR to assay the expression of IL-1β, IL-6, and TNF-α. The expression levels of these three cytokines were analyzed at each time point and are expressed as the fold change relative to the mRNA level in control cells exposed to PBS only. Data are representative of at least three independent experiments. *p≤0.05, **p≤0.01, ***p≤0.001.

We investigated whether knockout of PrP^C^ affected the expression of pro-inflammatory cytokines. Knockout of *PRNP* dramatically increased the expression of IL-1β, IL-6, and TNF-α during the entire experimental procedure, as revealed by qPCR (Figure 5A, B, and C). The variation tendency in cytokine expression was similar among PRNP^−/−^ and wild-type macrophages. However, the amplitude of variation of PRNP^−/−^ macrophages was higher than that of wild-type macrophages.

## Discussion

Macrophages act as the first line of protection in the innate immune response and play an important role in phagocytosis, antigen presentation, and inflammatory cytokine production. The classical activation of macrophages corresponds to the first phase, also known as the killing phase, of the innate immune response to acute stimuli and is characterized by the induction of a specific gene profile and the subsequent production of multiple cytoactive factors such as TNF-α, NO, and IL-1 that protect against tissue invaders [25]–[29]. A recent study performed in our laboratory showed that, in the long term, PrP^C^ may actively participate in the regulation of microglia during the activation process [30]. However, the role of PrP^C^ in the killing phase of macrophages has not been reported yet. Macrophages play an important role in facilitating the spread of prion infections from the periphery to the central nervous system [31], as prion protein normally is expressed on the surface of macrophages. To explore the role of PrP^C^ in macrophage phagocytosis, microbicidal activity, and activation, we chose EGFP-*E. coli* as a representative of general pathogenic microbe for infection of BMDMs. We found that *E. coli* infection altered the mRNA expression of *PRNP*. It is possible that upregulation of *PRNP* expression interferes with BMDM activation, suggesting a possible role of PrP^C^ in the host immune response. This observation is consistent with the effects of *Mycobacterium bovis* infection in BV2 microglia but differs from the findings of studies reporting exposure of microglia to interferon (IFN)-γ, IL-4, or IL-10 [15], [30]. This discrepancy may arise from the nature of bacterial infections, which are different from and more complex as a model than cytokine stimulation [15].

Because of the diversity among the bacteria, particles, cells, and methods used for the different experiments, reports on the relationship between PrP^C^ and phagocytic ability are controversial. Moreover, distinct rates of phagocytosis cannot be attributed to random variations as a result of mixed genetic backgrounds [14]. In a study on *M. bovis* infection, *PRNP* silencing did not affect the number of viable bacilli in infected microglia [15]. Additionally, PrP^C^ deficiency has been found to prevent swimming internalization of *Brucella abortus* into macrophages [32]. More efficient phagocytosis of zymosan particles was observed in ZrchI Prnp^−/−^ mice than in Prnp^+/+^ mice [14]. Rikn Prnp^−/−^ cells showed lower phagocytic activity than Prnp^+/+^ cells following ingestion of fluorescent beads [13], [33]. However, our results showed that PrP^C^ exerted a negative regulatory function in phagocytosis during *E. coli* infection, which is consistent with a previously reported in vivo assay [14].

Phagosome maturation into the phagolysosome is the innate immune defense mechanism of macrophages. In the absence of PrP^C^, the mRNA expression of *Rab5*, *Rab7*, and *Eea1* in BMDMs increased after infection with *E. coli*. Furthermore, increased recruitment of LAMP2 to phagosomes was observed, indicating that PrP^C^ played a negative regulatory role in phagosome maturation. These observations prompted us to hypothesize that PRNP^−/−^ macrophages formed phagosomes with an enhanced capacity to kill intracellular *E. coli*. Bacterial proliferation is the product of both replication and killing in the population as a whole [34]. Our data show that the number of CFUs increased in both wild-type and knockout macrophages, which may be explained by the observation that *E. coli* replicates inside macrophages and that the number of bacteria was higher than the clearance capacity of macrophages. However, the bacterial proliferation index observed in PRNP^−/−^ macrophages was much lower than that in wild-type macrophages. Therefore, PRNP^−/−^ macrophages were more efficient at bacterial clearance than wild-type macrophages, which indicates that PrP^C^ plays a negative regulatory role in phagosome maturation. To our knowledge, this is the first evidence that PrP^C^ has a negative effect on the defense function of macrophages in the killing phase.

Several lines of evidence indicate that NF-κB activation is critical for the induction of iNOS and the upregulation of inflammatory cytokines such as IL-1β, IL-6, and TNF-α [35]–[37]. Our previous study showed that macrophages exposed to neurotoxic prion peptides were activated through the activation of NF-κB [38]. In this study, we examined the effect of *E. coli* infection on the expression of the pro-inflammatory cytokines IL-1β, IL-6, and TNF-α in *PRNP*-knockout macrophages. Knockout of *PRNP* increased the expression of the pro-inflammatory cytokines, which would likely recruit more macrophages to inflammation sites and activate naïve macrophages to release ROS and active NO to kill the intracellular pathogens. Our CFU results for intracellular *E. coli* confirmed this effect. Moreover, recent studies have shown that recycling of endosomes is required for TNF-α trafficking. TNF-α is trafficked from the Golgi to recycling endosomes, which are delivered to the cell surface by the action of vesicle-associated membrane protein (VAMP) 3 [9]. In addition, IL-6 and IL-12 specifically induce the expression of *Rab5* and *Rab7* via the activation of extracellular signal-regulated kinase and p38 mitogen-activated protein kinase (MAPK), respectively, thereby modulating intracellular trafficking. Furthermore, TNF-α and IFN-γ significantly modify phagosome maturation [39]–[41]. These data suggest that cytokines can also modulate membrane trafficking, suggesting that PrP^C^ plays a role in this process.

A study by Aguzzi’s group provided a new perspective [42]. The theoretical basis of that study was that CD47 is a ligand for the extracellular region of signal regulatory protein alpha (SIRPα) and that the binding of CD47 on apoptotic cells to SIRPα on macrophages transmits a “don’t eat me” signal that protects the targets from phagocytosis [42]–[45]. CD47 is known to be a transmembrane protein that is ubiquitously expressed in host cells, but not in *E. coli* [43]. Therefore, our findings can be attributed mainly to PrP^C^ and not SIRPα. Accumulating evidence and the present study suggest that many other molecules play important roles in phagocytosis and cytokine secretion through different signaling pathways; such molecules include the soluble N-ethylmaleimide-sensitive factor attachment protein receptors (SNAREs), synaptotagmins (syts), Toll-like receptors (TLRs), Src family kinases, and cAMP [5], [9], [46]–[50]. Thus, although many studies have investigated the roles of PrP^C^, further studies are needed to clarify the relationships between PrP^C^ and these molecules.

In summary, our results revealed that in the acute phase of macrophage activation after infection by *E. coli*, PrP^C^ actively participates in the regulation of the process by protecting against excess inflammation through negative regulation of phagocytosis, phagosome maturation, cytokine expression, and microbicidal activity, which provides new insights into the physiological functions of PrP^C^ in macrophages. Further studies are necessary to determine how PrP^C^ regulates vesicular trafficking associated with phagocytosis and cytokine secretion.
